# Declining Medicare reimbursement and income in primary total shoulder arthroplasty: a comparison of osteopathic and allopathic orthopedic surgeons, 2013-2023

**DOI:** 10.1016/j.jsea.2026.100002

**Published:** 2026-01-29

**Authors:** Anthony J. Minerva, John W. Moore, Annie Jinks, Jason Silvestre, Brandon L. Rogalski, Richard J. Friedman

**Affiliations:** Department of Orthopaedics & Physical Medicine & Rehabilitation, Medical University of South Carolina, Charleston, SC, USA

**Keywords:** Shoulder, Arthroplasty, Reimbursement, Medicare, Utilization, Osteopathic, Allopathic

## Abstract

**Background:**

Medicare reimbursement for arthroplasty, including total shoulder arthroplasty (TSA), has declined over the past decade. The purpose of this study is to determine whether Medicare reimbursement differed between osteopathic (DO) and allopathic (MD) orthopedic physicians performing primary TSA and how this has changed over time.

**Methods:**

The Centers for Medicare and Medicaid Services databases were queried from 2013 to 2023 to identify physicians billing the Current Procedural Terminology code 23472 (primary TSA). Physicians were stratified by degree (12,518 MD vs. 1,167 DO). All monetary values were adjusted to 2023 United States dollars using the Consumer Price Index. Procedural reimbursement was defined as the Centers for Medicare and Medicaid Services–reported average Medicare standardized amount. Annual procedural income (API) was a physician's Medicare income from TSA (Current Procedural Terminology code 23472), calculated as average Medicare standardized amount multiplied by services billed.

**Results:**

In 2013, DO physicians earned 31% ($11,493) less in API than MD physicians ($25,704 vs. $37,197; *P* < .001), with 24% fewer mean years in practice (14 vs. 19; *P* < .001) and 30% fewer mean services (18 vs. 25; *P* < .001). In 2023, DO physicians earned 26% ($8,422) less in API than MD physicians ($24,001 vs. $32,423; *P* < .001), with 21% fewer mean years in practice (18 vs. 22; *P* < .001) and 25% fewer mean services (22 vs. 29; *P* < .001). From 2013 to 2023, the proportion of DO providers doubled (5% vs. 10%), as did their share of TSA services (4% vs. 8%). Over this time, procedural reimbursement did not differ significantly between degree types. However, DO physicians experienced a 23% ($335) decline in procedural reimbursement ($1,444 vs. $1,109; *P* < .001), with no significant change in mean services (*P* = .073) or API (*P* = .524), while MD physicians experienced a 24% ($345) decline in procedural reimbursement ($1,458 vs. $1,113; *P* < .001) despite billing 15% more mean services (25 vs. 29; *P* < .001), resulting in a 13% ($4,774) decline in API ($37,197 vs. $32,423; *P* < .001).

**Conclusion:**

By 2023, MD physicians experienced a 13% decline in API compared with 2013 despite averaging 15% more services, highlighting a concerning trend of providers working more for less. Both MD and DO physicians had significant declines in procedural reimbursement over this period (24% and 23%, respectively) despite a 31% rise in inflation. Although procedural reimbursement did not differ between degree types in either year, DO physicians consistently billed 7 fewer services and earned $11,493 less in API in 2013 and $8,422 less in 2023.

Health care expenditures in the United States continue to consume a large proportion of the nation's gross domestic product, placing increasing economic strain on physicians and patients.^11^ As the largest payer of orthopedic surgical procedures, Medicare plays a central role in health care policy, particularly given the aging populace.^9^^,^^17^ In response to rising costs, reimbursement models have sought to shift from a fee-for-service to a value-based care model, highlighted by the implementation of the Medicare Access and Children's Health Insurance Program Reauthorization Act in 2015 and the repeal of the sustainable growth rate.^13^ These changes introduced new reimbursement paradigms aimed at incentivizing high-quality care while minimizing cost.^21^

Despite these efforts, Medicare reimbursement for orthopedic procedures has shown a persistent downward trend, even as inflation and cost of living continue to rise.^2^^,^^9^ Total shoulder arthroplasty (TSA), including both primary and revision procedures, is now the fastest growing arthroplasty procedure in the United States, with utilization increasing over 1,500% during the past 2 decades.^12^^,^^15^^,^^17^^,^^18^ However, inflation-adjusted Medicare reimbursements for these procedures declined significantly, with even greater reductions observed in revision surgeries. In addition, geographic disparities have been noted, with the highest reimbursement rates in the Northeast and West and the steepest decline is in the Midwest and South, where procedural volume and utilization are the highest.^6^^,^^28^ Despite policy makers' efforts to prioritize quality-based models, current data show an inverse correlation between Medicare reimbursement and both patient complexity and satisfaction.^12^

As the health care landscape has evolved, so too has the orthopedic workforce composition, notably with more osteopathic (DO) applicants to orthopedic surgery residency programs.^36^ This shift is can be attributed to the 5-year transition, initiated in 2015, toward a single accrediting system for US residencies. This merger, completed in 2020, involved the Accreditation Council for Graduate Medical Education, the American Osteopathic Association, and the American Association of Colleges of Osteopathic Medicine.^29^ While DOs historically favored primary care specialties, recent years have seen a growing number of osteopathic physicians pursuing surgical fields, including orthopedic surgery.^22^ Though osteopathic applicants still match into orthopedic surgery residency at lower rates than their allopathic peers, this gap is narrowing.^16^^,^^36^ Importantly, evidence increasingly supports comparable clinical outcomes between MD and DO surgeons, including no significant differences in 30-day mortality, readmission rates, or hospital length of stay.^20^

Given the ever-changing political and economic landscape, including the annual updates to Medicare payment schedules, a nuanced understanding of reimbursement trends is critical for preventing financial strain and ensuring equitable access to high-quality surgical care. While many influencing factors have been studied, no research to date has examined whether physician medical degree (MD vs. DO) is associated with differences in Medicare reimbursement. The purpose of this study is to assess whether physician medical degree, osteopathic (DO) or allopathic (MD), influences Medicare reimbursement for primary TSA as measured by average Medicare standardized amount (AMSA) and annual procedural income (API), and if this has changed over time. We hypothesize that no differences in reimbursement exist between MD and DO orthopedic surgeons.

## Methods

This retrospective cohort study compared DO and MD orthopedic surgeons performing primary TSA. This study was exempt from institutional review board approval by using a publicly available database with physician-level data and without patient identifiers. The Centers for Medicare and Medicaid Services (CMS) “Medicare Physicians and Other Practitioners—by Provider and Service” database and the “National Downloadable File” provider data catalog were used to collect physician information, including medical degree type, billed services, and Medicare reimbursement.^25^^,^^26^ The dataset was queried from 2013 to 2023 for TSA procedures using the Current Procedural Terminology (CPT) code 23472: “arthroplasty, glenohumeral joint; total shoulder (glenoid and proximal humeral replacement)”.^23^

Per CMS cell suppression policy, physicians must bill a CPT code 10 or more times to be included in the CMS “Medicare Physicians and Other Practitioners—by Provider and Service” database for that year.^27^ Of the 29,954 providers who billed TSA, 14,075 were identified with “orthopedic surgery” as their primary specialty after merging the CMS databases using the National Provider Identifier as the key variable. After excluding nonphysicians (eg, physician assistants, nurse practitioners) and other non-DO/MD credentials (eg, MBBS), 13,685 physicians were stratified into MD (n = 12,518) and DO (n = 1,167) groups.

Physician characteristics and reimbursement data were compared between MD and DO physicians in 2013 and 2023 and within each degree type from 2013 to 2023. Physician characteristics included degree type and years in practice. Physicians were categorized into MD or DO according to the CMS “rendering provider credentials.” Years in practice were estimated by calculating the difference between the CMS reported medical school graduation year and the year the physician billed the indexed procedure.

Reimbursement data in this study included total services, mean services, procedural reimbursement, and API. Total services represent the total number of procedures billed by a provider for a given Healthcare Common Procedure Coding System code.^24^ Mean services were calculated as the average total services for DO and MD physicians. Monetary values were adjusted to 2023 United States dollars using the Consumer Price Index (CPI) from the U.S. Bureau of Labor Statistics.^30^ Procedural reimbursement was defined as the CMS-reported AMSA, a standardized payment metric that facilitates national-level comparisons independent of geographic payment adjustment factors.^19^^,^^24^ API was defined as a physician's Medicare income from TSA (CPT-23472), estimated as a physician's AMSA multiplied by their services billed.

Statistical analyses were performed using IBM SPSS Statistics, version 30.0.0.0 (IBM, Armonk, NY, USA) and R statistical software, version 4.3.2 (R Foundation for Statistical Computing, Vienna, Austria). Figures were generated using Microsoft Excel, version 16.97.2 (Microsoft Corporation, Redmond, WA, USA). Averages were reported as means. Continuous variables were compared using independent sample *t*-tests. Levene test was used to ensure equality of variances. Initial significance was defined with an alpha value of 0.05.

## Results

Physician characteristics and reimbursement data were compared in 2013 for DO physicians vs. MD physicians. (Table I). In 2013, DO physicians accounted for 5% of physicians who billed for TSA, performing 4% of the total services. DO physicians in 2013 averaged 24% fewer years in practice (14 vs. 19 years; *P* < .001), billed 30% fewer mean services (18 vs. 25; *P* < .001) than MD physicians, and earned 31% less in API than MD physicians ($25,704 vs. $37,197; *P* < .001). There was not a significant difference in procedural reimbursement in 2013 between DO physicians and MD physicians ($1,444 vs. $1,458; *P* = .760). In 2023, DO physicians accounted for 10% of physicians who billed for TSA, performing 8% of the total services. In 2023, DO physicians averaged 21% fewer years in practice (18 vs. 22; *P* < .001), billed 25% fewer mean services (22 vs. 29; *P* < .001) than MD physicians, and earned 26% less in API than MD physicians ($24,001 vs. $32,423; *P* < .001). There was not a significant difference in procedural reimbursement in 2023 between DO physicians and MD physicians ($1,109 vs. $1,113; *P* = .377).

**Table I:** Differences in physician characteristics and Medicare reimbursement data for DO physicians compared to MD physicians in 2013 and 2023.

Study variables	2013				2023			
	DO	MD	% difference	*P* value	DO	MD	% difference	*P* value
Physicians	35 (5%)	647 (95%)	–	–	189 (10%)	1,669 (90%)	–	–
Total services	622 (4%)	16,377 (96%)	–	–	4,124 (8%)	48,403 (92%)	–	–
Years in practice	14	19	−24	**<.001**∗	18	22	−21	**<.001**∗
Mean services	18	25	−30	**<.001**∗	22	29	−25	**<.001**∗
Procedural reimbursement	$1,444	$1,458	−1	.760	$1,109	$1,113	−0.3	.377
API	$25,704	$37,197	−31	**<.001**∗	$24,001	$32,423	−26	**<.001**∗

*DO*, osteopathic; *MD*, allopathic; *API*, annual procedural income.

∗ Indicates a statistically significance *P* value.

**Figure 1:**
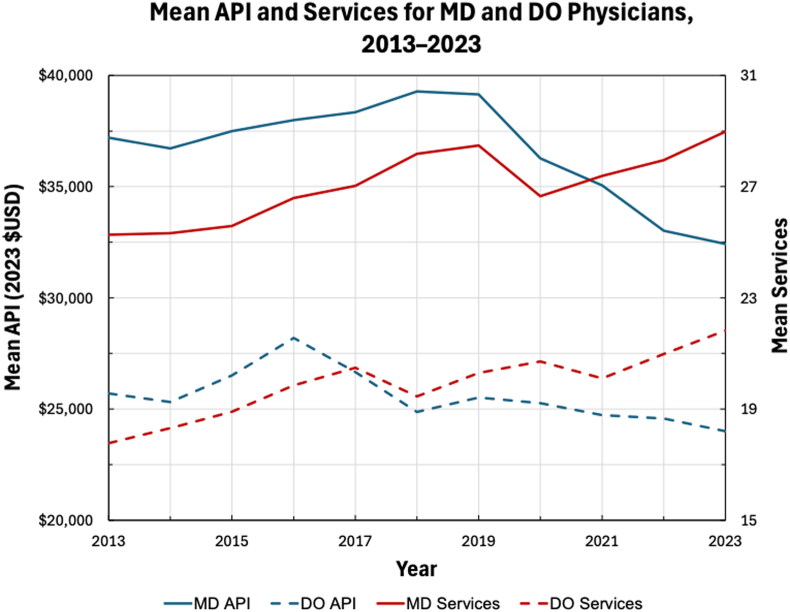
Comparison of mean API and mean services for DO and MD physicians from 2013 to 2023. *API*, annual procedural income; *USD*, United States dollar.

The change in physician characteristics and reimbursement data for DO physicians between 2013 and 2023 were compared. (Table II). From 2013 to 2023, there was a 440% increase in the number of DO physicians and a 563% increase in total DO-billed TSA services. DO physicians in 2023 averaged 22% more years in practice (14 vs. 18; *P* = .009) than DO physicians in 2013 and experienced a 23% decrease in procedural reimbursement compared to DO physicians in 2013 ($1,444 vs. $1,109; *P* < .001). There was not a significant change in mean services (18 vs. 22; *P* = .073) or API ($25,704 vs. $24,001; *P* = .524) for DO physicians in 2023 compared to 2013. For MD physicians between 2013 and 2023, there was a 158% increase in the number of MD physicians and a 196% increase in total MD-billed TSA services. MD physicians in 2023 averaged 18% more years in practice (19 vs. 22; *P* < .001) than MD physicians in 2013 and experienced a 24% decrease in procedural reimbursement compared to MD physicians in 2013 ($1,458 vs. $1,113; *P* < .001). Despite MD physicians in 2023 billing 15% more mean services (25 vs. 29; *P* < .001) than MD physicians in 2013, the API for MD physicians in 2023 was 13% less than the API for MD physicians in 2013 ($37,197 vs. $32,423; *P* < .001) (Fig. 1).

**Table II:** Changes in physician characteristics and Medicare reimbursement data for MD and DO physicians from 2013 to 2023.

Study variables	DO % change (2013 vs. 2023)	*P* value	MD % change (2013 vs. 2023)	*P* value
Physicians	+440% (35 vs. 189)	–	+158% (647 vs. 1,669)	–
Total services	+563% (622 vs. 4,124)	–	+196% (16,377 vs. 48,403)	–
Years in practice	+22% (14 vs. 18)	**.009**∗	+18% (19 vs. 22)	**<.001**∗
Mean services	+23% (18 vs. 22)	.073	+15% (25 vs. 29)	**<.001**∗
Procedural reimbursement	−23% ($1,444 vs. $1,109)	**<.001**∗	−24% ($1,458 vs. $1,113)	**<.001**∗
API	−7% ($25,704 vs. $24,001)	.524	−13% ($37,197 vs. $32,423)	**<.001**∗

*DO*, osteopathic; *MD*, allopathic; *API*, annual procedural income.

∗ Indicates a statistically significance *P* value.

## Discussion

While prior studies have demonstrated declining procedural reimbursement for TSA, no studies to date have examined the influence of physician degree type on differences in procedural reimbursement or rates of decline.^3^^,^^8^^,^^12^^,^^28^^,^^32^ Between 2013 and 2023, procedural reimbursement declined by 23% for DO physicians and 24% for MD physicians, despite a 31% rise in inflation. These findings reflect insufficient inflation adjustment from Medicare, which failed to keep pace with rising costs of living. Consequently, both DO and MD physicians were reimbursed substantially less per TSA procedure in 2023 than in 2013. For MD physicians, this decline translated into an imbalance between workload and compensation, despite performing 15% more mean services, their API decreased by 13%. Thus, MD physicians in 2023 not only earned less per TSA procedure but also earned lower total API than a decade earlier, despite performing more procedures. DO physicians experienced similar declines in reimbursement, while changes in mean services and API were not statistically significant, which may reflect the smaller sample size. Together, these trends highlight how increased procedural volume has been insufficient to offset declining reimbursement, leaving physicians compensated less for greater productivity.

Although procedural reimbursement did not differ significantly between DO and MD physicians in either 2013 or 2023, DO physicians consistently earned substantially less in API. This disparity was driven primarily by lower service volume, as DO physicians billed 30% fewer mean services in 2013 and 25% fewer in 2023 compared with MD physicians. Consequently, despite receiving essentially identical reimbursement per procedure, DO physicians earned 31% less in API in 2013 and 26% less in 2023. These findings suggest that API disparities were attributable primarily to procedural volume rather than inequities in Medicare reimbursement rates. Because standardized per-procedure reimbursement did not differ by degree type, observed differences in API should be interpreted as volume-based rather than reimbursement-based. Importantly, this study was not designed to identify causal mechanisms underlying differences in procedural volume, which may be influenced by factors not captured in CMS data, including referral patterns, case mix, subspecialization or fellowship training, practice model (academic vs. private), and institutional resources.

DO physicians demonstrated fewer years in practice compared with MD physicians; however, the present study cannot determine whether practice experience independently influences procedural volume. In this study, DO physicians consistently demonstrated fewer years in practice than MD physicians, with the gap narrowing slightly from averaging 24% fewer in 2013 to 21% fewer in 2023. Notably, this reduction in the practice experience gap paralleled decreases in service and income disparities: in 2013, DO physicians averaged 30% fewer mean services and 31% lower API than MD physicians, whereas by 2023, these gaps had narrowed to 25% and 26%, respectively. These findings support prior studies showing physicians earlier in their careers tend to perform fewer services and are more likely to receive lower annual earnings than colleagues with longer practice experience.^1^^,^^4^^,^^5^^,^^14^^,^^35^

The persistent but narrowing gap in practice experience between DO and MD physicians may be partially explained by the rapid expansion of the DO orthopedic workforce. Between 2013 to 2023, the number of DO surgeons billing TSA increased by 440% compared with a 158% increase for MD physicians, and the proportion of DO physicians participating in TSA billing doubled from 5% to 10%. Similarly, DO-billed TSA services increased 563% compared with 196% for MD physicians, doubling the DO contribution of total TSA services from 4% in 2013 to 8% in 2023. These trends highlight not only the growth of the DO workforce but also their increasing contribution to the TSA workload. The expansion of DO medical schools and greater acceptance of DO applicants into orthopedic residency programs likely contributed to these increases.^22^^,^^33^

To our knowledge, this is the first study comparing Medicare reimbursement rates for DO and MD orthopedic physicians performing TSA, and the largest study analyzing physician procedural reimbursement decline in TSA to date. Although other papers have attributed differences in reimbursement to geographic practice location, this study accounted for geographic differences by using AMSA for provider reimbursement.^7^^,^^10^^,^^12^^,^^28^^,^^31^^,^^34^ Utilization of AMSA allowed systemic differences in reimbursement to be assessed between physicians, as the CMS standardized reimbursement across physicians by removing extrinsic factors, including local wage indexes and incentive payment bundles.^19^ Limitations for this study include those inherent to database studies, including human error in data input and coding errors. The CMS database only captures Medicare reimbursement, meaning these results are not generalizable to total income for any provider that also accepts reimbursement from other payers. Due to Health Insurance Portability and Accountability Act regulations and CMS cell suppression policy, physicians who billed a service fewer than 10 times are excluded from the dataset, potentially underestimating the number of low-volume surgeons. Physicians not listed in the National Downloadable File were also excluded. In addition, API was estimated by multiplying a physician's inflation-adjusted AMSA by their total billed services; this value is not a direct measure of actual physician income. The CMS public-use files do not capture surgeon fellowship training, subspecialization, referral patterns, or practice model (academic vs. private), precluding stratified analyses based on these characteristics. Accordingly, differences in procedural volume observed in this study should be interpreted as associations rather than causal effects.

## Conclusion

By 2023, MD physicians experienced a 13% decline in API compared with 2013 despite averaging 15% more services, highlighting a concerning trend of providers working more for less. Both MD and DO physicians had significant declines in procedural reimbursement over this period (24% and 23%, respectively) despite a 31% rise in inflation. Although procedural reimbursement did not differ between degree types in either year, DO physicians consistently billed 7 fewer services and earned $11,493 less in API in 2013 and $8,422 less in 2023.

## Disclaimers

Funding: None.

Conflicts of interest: The authors, their immediate families, and any research foundation to which they are affiliated have not received any financial payments or other benefits from any commercial entity related to the subject of this article.
